# Diet and environment drive the convergence of gut microbiome in wild-released giant pandas and forest musk deer

**DOI:** 10.1016/j.isci.2025.112837

**Published:** 2025-06-07

**Authors:** Chenyi Gao, Qiuyu Huang, Xinru Yang, Xinyuan Cui, Kaizhi Wen, Yue Liu, Chenyan Wang, Qinlong Dai, Jiadong Xie, Lifeng Zhu

**Affiliations:** 1 School of Medicine, Nanjing University of Chinese Medicine, Nanjing 210023, China; 2 Key Laboratory of Drug Target and Drug for Degenerative Disease, Nanjing University of Chinese Medicine, Nanjing 210023, China; 3 Sichuan Liziping National Nature Reserve, Shimian, China; 4 Shimian Research Center of Giant Panda Small Population Conservation and Rejuvenation, Shimian, China; 5 School of Artificial Intelligence and Information Technology, Nanjing University of Chinese Medicine, Nanjing, Jiangsu Province, China; 6 Jiangsu Province Engineering Research Center of TCM Intelligence Health Service, Nanjing, Jiangsu Province, China

**Keywords:** Nature conservation, Zoology, Microbiology

## Abstract

Reintroduction is important for recovering endangered species, and gut microbiome is crucial for successful wildlife reintroduction. This study utilized 16S rRNA high-throughput sequencing of 791 fecal samples to examine the gut microbial changes in giant pandas (*Ailuropoda melanoleuca*) and forest musk deer (*Moschus berezovskii*) across captivity, semi-release, and release stages. Our results revealed a similar transitional pattern in the gut microbiome of both species, with semi-release stage displaying an intermediate state between captive and wild microbiome. We also observed that both species are enriched in *Pseudomonas* and functional pathways related to amino acid metabolism, ATP-binding cassette transporters, and acetyl-CoA/propionyl-CoA carboxylase. Furthermore, the SourceTracker analysis indicated putative contributions of plant and soil microbiome to the gut microbiome of forest musk deer. These findings suggest that similar herbivorous diets and same environment may contribute to the convergence of gut microbiome. In conclusion, our study provides valuable insights for reintroducing endangered wildlife.

## Highlights

• Convergence of gut microbial were detected in giant pandas and forest musk deer
• There were transition states and enriched *Pseudomonas* in both two species
• Putative contribution of environment was found to forest musk deer’s gut microbiome

## Graphical abstract

**Figure:**
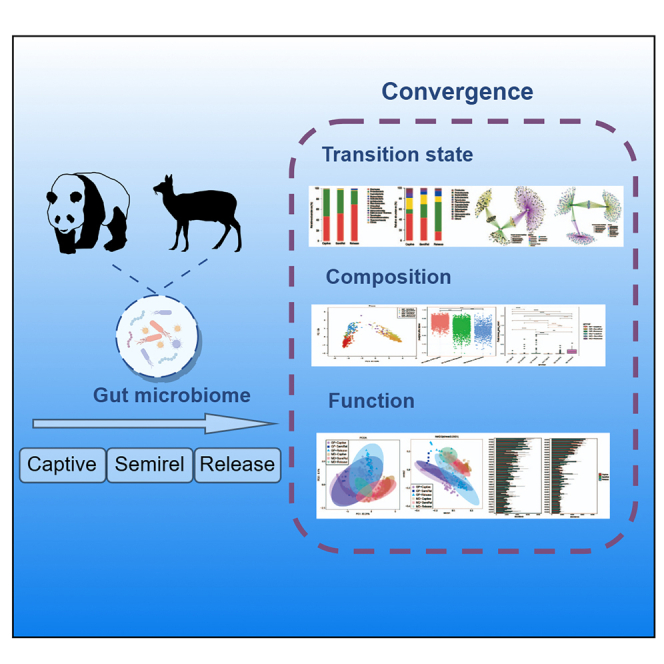

## Introduction

The gut microbiome plays a crucial role in host adaptation through intricate host-microbe interactions. It has been shown that the gut microbiome in reintroduced individuals significantly contributes to host adaptive evolution, nutrition, disease resistance, and ecological adaptation.^1^^,^^2^^,^^3^^,^^4^ The composition of the gut microbiome is primarily shaped by host phylogeny and diet, while environmental conditions and social behaviors also play a role in influencing the gut microbiome.^5^^,^^6^^,^^7^^,^^8^^,^^9^ In addition, diet and environment can overtake host phylogeny, driving convergent evolution in gut microbial composition and function. It has been shown that bamboo specialists from two different mammalian orders (primates and carnivora) share a high number of low-abundance gut microbiome.^10^ Similarly, the gut microbiome of yaks and Tibetan sheep has exhibited functional convergence to adapt to energy metabolism in the same high-altitude environments.^11^ Furthermore, Song et al. reported a dominance of *Proteobacteria* in the gut microbiome of bats and birds, likely driven by flight-associated metabolic demands.^12^ These researches mainly focused on the phenomena and driving factors of gut microbial composition and function convergence, elucidating the synergistic evolutionary relationship between host and gut microbiome. However, our work focused on wildlife gut microbial convergence and applied it to wildlife conservation, offering critical guidance for conservation strategies and reproductive management.

Conservation metagenomics is an emerging branch of conservation biology that employs metagenomics technology to explore the composition, function, and host-microbe interactions of microbial communities.^13^ The application of conservation metagenomics in wildlife release programs can provide valuable insights into the ecology, evolution, and conservation of wildlife, as demonstrated in species, such as giant pandas,^13^^,^^14^ Amur tiger,^15^ camels,^16^ and Alpine musk deer.^17^ The giant pandas and forest musk deer are the world’s endangered animals.^18^^,^^19^ For endangered wildlife, rewilding is a key strategy for protecting wild populations, and substantial progress has been achieved in the rewilding and release of these species. For instance, Yao et al. identified that the gut microbiome of translocated giant pandas showed a transitional state, and the effectiveness of existing translocation strategies has been evaluated, providing a scientific foundation for wild release programs.^14^ Huang et al. further confirmed the transitional state of giant pandas from captivity to wild release, offering potential biomarkers to evaluate the success of reintroduction efforts in natural habitats.^13^ Consequently, monitoring gut microbiome dynamics during the wild release of endangered species enhances our understanding of the specific microbiological changes in released animals,^17^^,^^20^^,^^21^ thereby improving release success rates.

Rewilding is a vital strategy for revitalizing and rebuilding wild populations, significantly conserving endangered species. In this study, both giant pandas and forest musk deer underwent a progression from captivity to wilderness training, culminating in their release into a shared natural habitat. Building on this, conservation metagenomics was applied to address two primary objectives: (1) to investigate dynamic changes of the gut microbiome in giant pandas and forest musk deer across distinct stages—captive, semi-release, and release; and (2) to evaluate whether convergence in gut microbiome composition and function occurred between these two species after their release into the same habitat.

## Results

### Giant pandas and forest musk deer showed convergence of composition and a similar transition from captive to release

**Figure 1:**
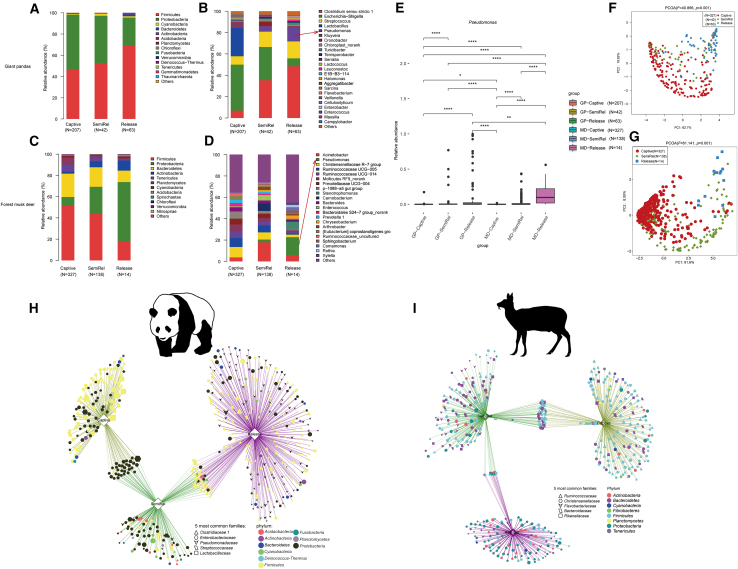
Transitional changes in the gut microbiome of giant pandas and forest musk deer from captivity to release N represents the number of samples at each stage (A and B) Relative abundances of the predominant bacterial phyla (A) and genera (B) in the gut microbiome of giant pandas at different stages. (C and D) Relative abundances of the predominant bacterial phyla (C) and genera (D) in the gut microbiome of forest musk deer at different stages. (E) Relative abundance of *Pseudomonas* across different groups at each stage, highlighting its enrichment in the release stage. Wilcoxon rank-sum test, ∗*p* < 0.05, ∗∗*p* < 0 0.01, and ∗∗∗∗*p* < 0.0001. (F) Principal coordinate analysis (PCoA) based on weighted UniFrac distances showing clustering patterns of gut microbiome in giant pandas across the three stages. Permutational multivariate analysis of variance (PERMANOVA) statistical analyses were conducted with 999 permutations using the function Adonis. (G) PCoA based on weighted UniFrac distances showing clustering patterns of gut microbiome in forest musk deer across the three stages. PERMANOVA statistical analyses were conducted with 999 permutations using the function Adonis. (H and I) Co-occurrence network analyses highlighting the connections of gut microbiome in giant pandas (H) and forest musk deer (I) at different stages, with major bacterial families and their phyla indicated.

**Figure 2:**
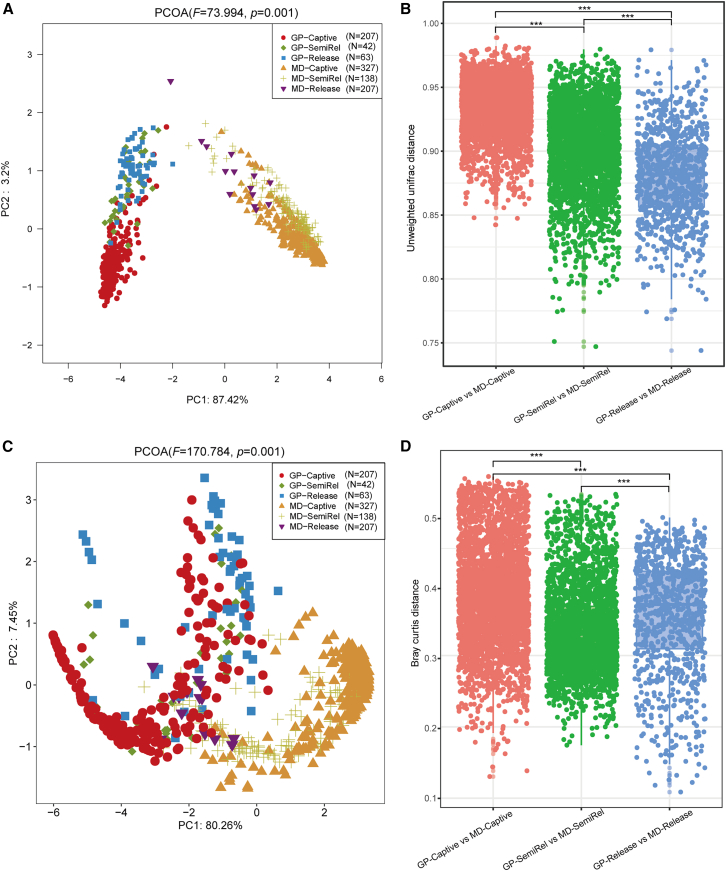
Microbial and functional convergence in the gut microbiome of giant pandas and forest musk deer N represents the number of samples at each stage. (A) Principal coordinate analysis (PCoA) based on unweighted UniFrac distances, illustrating gut microbiome composition across different groups. PERMANOVA statistical analyses were conducted with 999 permutations using the function Adonis. (B) Boxplot showing unweighted UniFrac distances to compare the gut microbiome composition between giant pandas (GP) and forest musk deer (MD) at captive, semi-release, and release stages. Student’s t test, ∗∗∗*p* < 0.001. (C) PCoA based on Bray-Curtis distances, illustrating functional differences at the COG (cluster of orthologous groups) level across groups. PERMANOVA statistical analyses were conducted with 999 permutations using the function Adonis. (D) Boxplot comparing Bray-Curtis distances of functional differences between giant pandas and forest musk deer across the three stages. Student’s t test, ∗∗∗*p* < 0.001.

**Figure 3:**
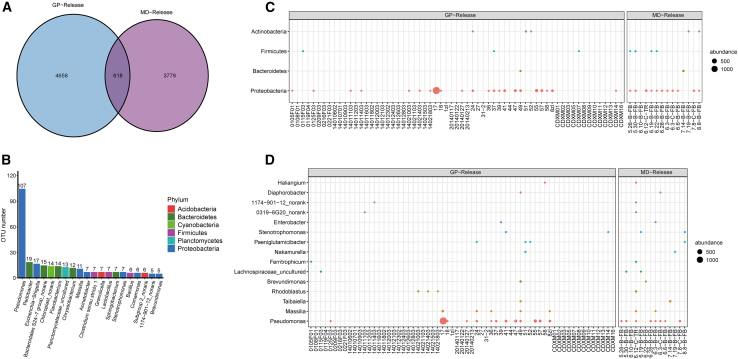
Shared OTUs analysis between released giant pandas and forest musk deer (A) Venn diagram illustrating the number of operational taxonomic units (OTUs) shared between the gut microbiome of released giant pandas (GP-Release) and forest musk deer (MD-Release). (B) TOP 20 genera of shared OTUs between released giant pandas and forest musk deer. (C) Classification of shared OTUs at the phylum level, with different colors representing microbial taxa and bubble size indicating relative abundance. (D) Classification of shared OTUs at the genus level, with different colors representing microbial taxa and bubble size indicating relative abundance.

Principal coordinate analysis (PCoA) (PERMANOVA, *F* = 73.994, *p* = 0.001) and comparative analysis based on unweighted UniFrac distances revealed a progressive convergence of the gut microbiome in the two species as the release process advanced. Specifically, the gut microbiome differences between giant pandas and forest musk deer were reduced in the release stage compared to the captive stage (Figures 2A and 2B). In both giant pandas and forest musk deer, we found an increased relative abundance of *Pseudomonas* after reintroduction (Figures 1B and 1D). For example, *Pseudomonas* was rarely found in captive giant pandas but showed an enhanced abundance upon their release into the wild. Similarly, wild-released forest musk deer demonstrated a significant increase in *Pseudomonas* abundance compared to the captive stage (Figure 1E). Additionally, Lefse (linear discriminant analysis effect size) identified an enrichment of *Pseudomonas* in both species (Figure S4). To further explore the convergence, shared operational taxonomic units (OTUs) were analyzed. A total of 618 OTUs were found to be shared between released giant pandas and forest musk deer (Figure 3A). These shared OTUs were primarily classified within the genus *Pseudomonas* (*Proteobacteria*) (Figures 3B–3D).

PCoA based on weighted UniFrac distances showed clear clustering patterns for each stage in giant pandas (PERMANOVA, *F* = 40.866, *p* = 0.001) and forest musk deer (PERMANOVA, *F* = 61.141, *p* = 0.001), with the semi-release stage serving as an evident transitional state (Figures 1F and 1G). Analyses of the gut microbiome at the phylum and family levels further supported this transitional characterization. The gut microbiome of the semi-release stage was a mixture of captive and release stages (Figures 1A, 1C, S1F, and S2F). Moreover, network analysis observed extensive microbiome interactions between the semi-release stage and both captive and release stages in giant pandas and forest musk deer (Figures 1I and 1J), which also indicated a transitioning state from captive to the wild during the semi-release stage.

### Functional convergence of released giant pandas and forest musk deer

**Figure 4:**
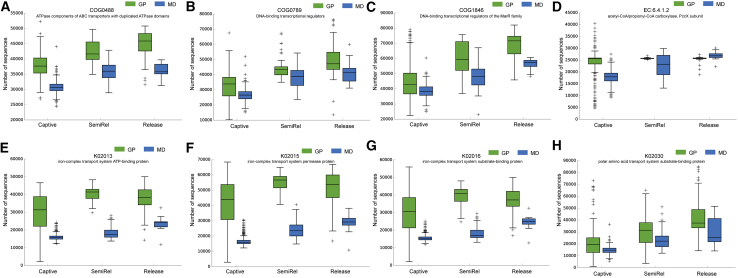
COG and KEGG enrichment analysis of functional features in giant pandas and forest musk deer Data are presented as the median value, lower quartile, and upper quartile, dots indicate discrete points. (A–C) Giant pandas and forest musk deer convergent function at the COG function level. (D) Giant pandas and forest musk deer convergent function at the KEGG enzyme function level. (E–H) Giant pandas and forest musk deer convergent function at the KEGG orthology function level.

To explore functional convergence, we performed Tax4Fun functional prediction on the gut microbiome of giant pandas and forest musk deer. PCoA based on Bray-Curtis distances at both the COG (cluster of orthologous groups of proteins) (PERMANOVA, *F* = 170.784, *p* = 0.001) and KEGG (Kyoto encyclopedia of genes and genomes) (PERMANOVA, *F* = 175.58, *p* = 0.001) levels revealed a gradual functional convergence as the release process advanced (Figures 2D and S3F). In addition, the comparative analysis based on Bray-Curtis distances at the function level also revealed a reduction in difference (Figures 2C and S3C). Analysis of the COG database revealed that both giant pandas and forest musk deer exhibited enrichment in DNA-binding transcriptional regulators of the MarR family (COG1846), ATPase components of ATP-binding cassette (ABC) transporters with duplicated ATPase domains (COG0488), and DNA-binding transcriptional regulators (COG0789) after release (Figures 4A–4C). Similarly, exploration using the KEGG orthology (KO) database showed enrichment in proteins associated with the iron-complex transport system, including permease proteins (K02015), ATP-binding proteins (K02013), and substrate-binding proteins (K02016), as well as polar amino acid transport system substrate-binding proteins (K02030) (Figures 4E–4H). These enriched proteins are primarily associated with the ABC transporter family. Additionally, enhancement was observed in the amino acid metabolism pathway (Figures S5A and S5E) and in the enzyme acetyl-CoA/propionyl-CoA carboxylase, PccX subunit (EC:6.4.1.2) (Figure 4D) in both released giant pandas and forest musk deer.

### Putative contributions of the living environmental microbiome to the forest musk deer

**Figure 5:**
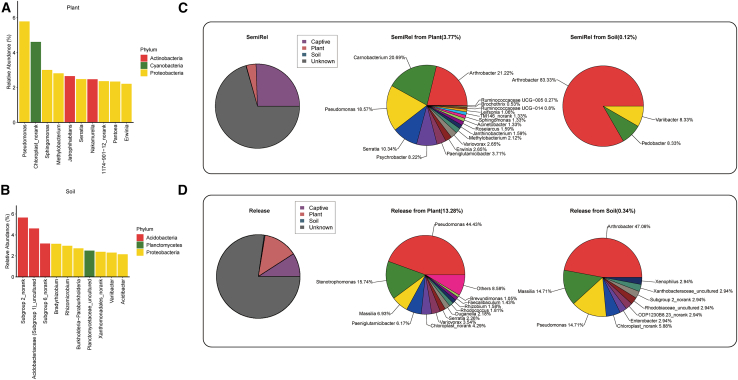
The SourceTracker analysis of gut microbiome in forest musk deer (A) Genera with top 10 abundance in plants. (B) Genera with top 10 abundance in soil. (C) SourceTracker analysis of gut microbiome in the semi-release forest musk deer and the percent of bacteria originating from plant and soil at the semi-release stage. (D) SourceTracker analysis of gut microbiome in released forest musk deer and the percent of bacteria originating from plant and soil at the released stage.

We analyzed the composition of plant and soil microbiome and found that the main microbial genera in plants were *Pseudomonas* and *Chloroplas_norank*, while *Subgroup 2_norank* (*Acidobacteria*) and *Acidobacteriaceae (Subgroup 1)_uncultured* were predominant in soil (Figures 5A and 5B|). SourceTracker analysis revealed the potential contribution of plant and soil microbiome to the gut microbiome of forest musk deer. In the semi-release stage, the putative contributions from plants and soil in forest musk deer were 3.77% and 0.12%, respectively. In contrast, for released forest musk deer, the putative contributions from plants and soil were 13.28% and 0.34%, which were higher than the semi-release stage. Furthermore, in the gut microbiome of released forest musk deer, further analysis indicated that 13.28% of the plant-derived microbial input was primarily attributable to *Pseudomonas*, with *Pseudomonas* species comprising 44.43% of this contribution. Additionally, in released forest musk deer, the plant microbiome contributed 0.34% to the gut microbiome, of which *Pseudomonas* accounted for 14.71% (Figures 5C and 5D).

## Discussion

### Convergence in the gut microbiome of giant pandas and forest musk deer after release into the same environment

Investigating changes in the gut microbiome during the stages of wild training and release provides valuable insights into the adaptation of wildlife to natural environments and the effectiveness of translocation efforts. Previous studies have revealed the transitional state of gut microbiome from captivity to wild release^14^ and identified three distinct gut microbiome profiles in giant pandas across the captive, wild training, and release stages, highlighting *Pseudomonas* as a potential biomarker for successful reintroduction into the wild.^13^ Other studies have demonstrated that the gut microbiome of captive wildlife (e.g., woolly monkeys and Przewalski’s horse) undergoes significant changes following release into natural habitats.^21^^,^^22^ Consistent with these findings, our study revealed a transitional status at the semi-release stage and enriched *Pseudomonas* after releasing into the same living environment. This transition state indicated the restoration of captive individuals’ wildness, reflecting adaptation to the translocation environment.

We also found enriched *Pseudomonas* in wild-released giant pandas and forest musk deer, with *Pseudomonas* as the first enriched genus among the 618 OTUs shared by the two groups (Figure 3B). Wild giant pandas predominantly consume bamboo, while wild forest musk deer feed on leaves from various high-fiber plants. *Pseudomonas* is recognized as a prominent symbiotic microbiota in plants, including bamboo.^23^ It has been shown that the gut microbiome of wild giant pandas has a high proportion of *Pseudomonas*, which correlates with the bamboo-based diet.^14^^,^^24^^,^^25^ Our SourceTracker analysis investigated that in the release stage of forest musk deer, the gut microbiome may be transferred from the plant and soil microbiome, with *Pseudomonas* being the predominant contributing species. Furthermore, plants can generate various secondary metabolic compounds like phenolic and terpenoid compounds,^26^ with *Pseudomonas* playing a crucial role in their degradation.^27^^,^^28^ And *Pseudomonas* is proven to be involved in the detoxification of cyanide in bamboo.^24^ Additionally, it has been found that a laccase lac51 in *Pseudomonas* can hydrolyze lignin (substances attached to cellulose and hemicellulose), which further facilitates the digestion of cellulose.^29^ Overall, the enriched *Pseudomonas* in giant pandas and forest musk deer suggested an adaptation to herbivorous diets and environments.

### Functional convergence in the gut microbiome of giant pandas and forest musk deer

Our findings also revealed functional convergence in the gut microbiome of giant pandas and forest musk deer, with both species showing enrichment in amino acid metabolic pathways, ABC transporter proteins, and biotin-dependent carboxylases such as acetyl-CoA carboxylase and propionyl-CoA carboxylase. Proteins derived from bamboo have been identified as a significant energy source for giant pandas.^30^^,^^31^ Wild pandas consume more bamboo than their captive counterparts, which may explain the observed enrichment in amino acid metabolic pathways. Similarly, nutrient composition analysis of plant leaves preferred by wild forest musk deer indicated high protein content.^32^ As protein is a key component of musk, the upregulation of amino acid metabolic pathways in wild forest musk deer likely reflected an adaptive response to their specific dietary and habitat conditions.

ABC transporter proteins, known for mediating the uptake of diverse nutrients—including mono- and oligosaccharides, organic and inorganic ions, amino acids, peptides, iron-siderophores, metals, polyamine cations, opines, and vitamins—were also enriched in the gut microbiome of both wild-released giant pandas and forest musk deer.^33^ This enrichment suggested that the gut microbiome of these species may optimize nutrient absorption through ABC transporter systems. Additionally, both species exhibited enrichment in biotin-dependent carboxylases, specifically acetyl-CoA carboxylase and propionyl-CoA carboxylase, which play essential roles in the metabolism of fatty acids, amino acids, and carbohydrates.^34^^,^^35^^,^^36^ These metabolic features likely represented adaptations to dietary changes associated with the rewilding process and could provide valuable insights into the physiological status and health of giant pandas and forest musk deer. However, our results were based on the functional prediction of Tax4Fun, which carries inherent limitations due to the lack of experimental validation of the predicted functions. Future studies will incorporate metagenomic sequencing and qPCR analyses to experimentally verify these functional predictions.

### Environmental microbial transmission might contribute to the enrichment of Pseudomonas

Environmental factors and diet are widely recognized as key determinants of gut microbiome composition. It has been shown that the marine microbiome may have colonized marine carnivores during evolution, leading to the convergence of gut microbiome in these animals.^37^ Moreover, the gut microbiome of yaks and Tibetan sheep have exhibited functional convergence to adapt to energy metabolism in the same high-altitude environments.^11^ In terms of diet, the high percentage of bamboo intake has been suggested as a key driver of gut microbial function convergence between giant pandas and Yunnan snub-nosed monkeys.^38^ Similarly, dietary influences have been reported to drive microbiome convergence between bamboo-eating pandas and certain invertebrate insects.^25^ Therefore, we speculated that the convergence of gut microbiome in giant pandas and forest musk deer may be influenced by the environment and diet.

The environmental microbiome may influence the host’s gut microbiome through microbial transmission. For instance, changes in the seasonal habitats of amphibians could lead to symbiotic microbiome carrying different origins (water or soil source) microbiome.^39^ Additionally, Zhu et al. found a high similarity between the gut microbiome communities of herbivorous insects and the dietary plants’ symbiotic microbiome, suggesting a probable microbial transmission originating from the diet.^40^ In our study, we identified the increased putative contributions of environmental microbiome (e.g., plant and soil source microbiome) to forest musk deer symbiotic microbiome from semi-release (3.89%) to release (13.62%) stages. We hypothesized that the environmental microbial transmission may result in gut microbial convergence (high abundance of *Pseudomonas*). *Pseudomonas* demonstrates efficient cellulose and lignin degradation capabilities,^41^^,^^42^ as well as putatively cyanogenic compound detoxification in the bamboo-eating panda’s gut.^24^ Environmentally acquired *Pseudomonas* may contribute to dietary adaptation mechanisms in giant pandas and forest musk deer.

These findings are important for understanding how the living environments affect host-microbiome relationships and how the gut microbiome contributes to convergent evolution in phylogenetically divergent species with shared habitats and herbivorous diets. The convergence of gut microbiome between giant pandas and forest musk deer may expand their dietary ecological niches. In addition, it may influence local soil and environmental microbiome, leading to widespread transmission of *Pseudomonas*-associated mobile genetic elements (MGEs) and antibiotic resistance genes (ARGs) between animals and the environment. Our findings indicate that before the implementation of giant pandas and forest musk deer release programs, it is critical to conduct comprehensive habitat assessments, including food resource availability and potential environmental risk factors. Furthermore, establishing ARGs flow surveillance networks is essential to monitor the transmission dynamics of pathogens and antimicrobial resistance genes across environmental reservoirs and host species.

### Conclusion

In this study, we revealed that the gut microbiome of both species exhibited a similar transitional state from captivity to the wild. We observed microbial and functional convergence in the gut microbiome of giant pandas and forest musk deer. Moreover, SourceTracker analysis suggested that the gut microbiome of forest musk deer may be influenced by environmental microbial transmission from plants and soil, which may explain the enriched *Pseudomonas*. We speculated that this convergence may be driven by their shared habitat environment and plant-based diet. These findings provide insights into the ecological adaptation of gut microbiome during rewilding by highlighting the influence of diet and habitat in shaping microbial communities. In doing so, this research advances our understanding of the role of gut microbiome in facilitating wildlife release and contributing to conservation biology.

### Limitations of the study

Functional level analyses in this study were mainly based on 16S rRNA sequencing for functional prediction, and no deeper functional analyses of metagenomics were performed. Future studies could employ metagenomic approaches to validate the expression profiles of key cyanide detoxification-related genes in giant pandas and forest musk deer, including rhodanese (thiosulfate: cyanide sulfurtransferase, EC 2.8.1.1), nitrilase (Nit, EC 3.5.5.1), and cob (I) alamin adenosyltransferase (EC 2.5.1.17).^24^^,^^43^ Additionally, future studies could assess ligninocellulose-degrading enzymes (e.g., glycoside hydrolases and laccases^44^) and functional enrichment analyses in amino acid metabolism, ABC transporter activity, and acetyl-CoA/propionyl-CoA carboxylase-mediated pathways to further elucidate the metabolic adaptations to their specialized herbivorous diets.

## Resource availability

### Lead contact

Requests for further information and resources should be directed to and will be fulfilled by the lead contact, Lifeng Zhu (zhulf2020@126.com).

### Materials availability

This study did not generate new, unique reagents.

### Data and code availability

• The giant pandas’ data are publicly available as of the date of publication at https://doi.org/10.1016/j.gecco.2019.e00644. The forest musk deer, plant, and soil data have been deposited at NCBI: PRJNA665181 and PRJNA1222155. The silhouettes of giant pandas (*Ailuropoda melanoleuca*) and forest musk deer (*Moschus berezovskii*) are from PhyloPic (http://phylopic.org/); both are available under a CC0 1.0 Universal Public Domain Dedication (https://creativecommons.org/publicdomain/zero/1.0/).
• This paper does not report original code.
• Any additional information required to reanalyze the data reported in this paper is available from the lead contact upon request.

## Acknowledgments

This research was funded by the 10.13039/501100001809National Natural Science Foundation of China (grant number 32270546) and the 10.13039/501100012246Priority Academic Program Development of Jiangsu Higher Education Institutions.

## Author contributions

Conceptualization and methodology, L.Z.; investigation, C.G., Q.H., X.Y., X.C., K.W., and C.W.; formal analysis, C.G., X.C., Q.D., Y.L., and J.X.; writing—original draft, C.G.; writing—review and editing, Q.D., and L.Z.

## Declaration of interests

The authors declare no competing interests.

## STAR★Methods

### Key resources table

**Table:** 

REAGENT or RESOURCE	SOURCE	IDENTIFIER
**Biological samples**		

Giant pandas	Yao et al.^14^	https://doi.org/10.1016/j.gecco.2019.e00644
Forest musk deer	Li. et al.^9^	NCBI: PRJNA665181
Forest musk deer, plant, and soil	This study	NCBI: PRJNA1222155

**Critical commercial assays**		

Fast DNA SPIN Kit for Feces	MPBIO	Cat#MP116570200
E. Z.N.A. Soil DNA Kit	Omega Bio-tek	D5625-02

**Deposited data**		

Sequencing data	This study	NCBI: PRJNA1222155

**Software and algorithms**		

Trimmomatic v0.33	http://www.usadellab.org/cms/?page=trimmomatic	http://www.usadellab.org/cms/?page=trimmomatic
cutadapt v1.9.1	https://github.com/marcelm/cutadapt	https://github.com/marcelm/cutadapt
FLASH v1.2.7	https://ccb.jhu.edu/software/FLASH/index.shtml	https://ccb.jhu.edu/software/FLASH/index.shtml
UCHIME v4.2	http://drive5.com/usearch/manual/uchime_algo.html	http://drive5.com/usearch/manual/uchime_algo.html
UCLUST v1.2.22q	https://www.drive5.com/usearch/manual/uclust_algo.html	https://www.drive5.com/usearch/manual/uclust_algo.html
Mothur v1.30.1	https://mothur.org/	https://mothur.org/
QIIME v1.7.0	http://qiime.org/index-qiime1.html	http://qiime.org/index-qiime1.html
R v4.1.0	https://www.r-project.org/	https://www.r-project.org/
Draw Venn Diagrams	http://bioinformatics.psb.ugent.be/webtools/Venn	http://bioinformatics.psb.ugent.be/webtools/Venn

### Method details

#### Sample collection and processing

Fecal samples were collected from two endangered species, giant pandas (*Ailuropoda melanoleuca*) and forest musk deer (*Moschus berezovskii*) at three distinct stages: captive, semi-release, and release. For giant pandas, 207 fecal samples were collected during the captive stage, 42 during the semi-release stage, and 63 during the release stage. In the case of forest musk deer, 327 fecal samples were collected during captivity, 138 during semi-release, and 14 during release. The released forest musk deer were monitored via GPS telemetry, a methodology challenged by signal attenuation in complex montane habitats, resulting in a constrained sample size. All captive samples were obtained from the Chengdu Research Base of Giant Panda Breeding, semi-release and release stage samples were collected from individuals which had undergone rewilding within the Liziping Natural Reserve in Sichuan Province, China. Sampling took place over three years, from 2018 to 2020, to capture microbial variation across the different stages of captivity, semi-release, and full release into the wild. Additionally, we collected 21 plant samples and 23 soil samples from the forest musk deer habitat. Sample information on giant pandas at all stages and forest musk deer in captive stages was derived from previously published data.^9^^,^^12^ All samples were immediately placed in sterile containers and stored at −80°C to preserve microbial DNA for subsequent analysis. Detailed information regarding the number of samples collected at each stage for both species is provided in Table S1, and detailed information on the plants and soil samples is provided in Table S2.

#### DNA extraction and 16S rRNA gene sequencing

Fecal samples of giant pandas and forest musk deer were soaked in anhydrous ethanol, and then the precipitate was enriched for subsequent DNA extraction. Total DNA was extracted using the Fast DNA SPIN Kit for Feces (MPBIO, CA, USA), following the manufacturer’s protocol to optimize DNA yield and purity. The DNA from soil and plants was extracted by the E. Z.N.A. Soil DNA Kit (Omega Bio-Tek, Norcross, GA, USA), following the manufacturer’s protocols. The quality and quantity of extracted DNA were assessed using a NanoDrop ND-2000 spectrophotometer (Thermo Fisher Scientific, USA) and agarose gel electrophoresis. Samples with high-quality DNA (A260/A280 ratio of ∼1.8) were selected for downstream analyses. The V4-V5 hypervariable region of the bacterial 16S rRNA gene was amplified using the primer pair 515F (5′-barcode-GTGCCAGCMGCCGCGG-3′) and 907R (5′-CCGTCAATTCMTTTRAGTTT-3′). Each primer included a unique 8 bp barcode to enable multiplexing of samples. PCR reactions were performed in a total volume of 25 μL containing 1× Phusion High-Fidelity PCR Master Mix (New England Biolabs, USA), 0.2 μM of each primer, and ∼10 ng of template DNA. The thermocycling conditions were as follows: an initial denaturation at 95°C for 2 min; 25 cycles of denaturation at 95°C for 30 s, annealing at 55°C for 30 s, and extension at 72°C for 45 s; followed by a final extension at 72°C for 5 min. Negative controls (no template DNA) were included in each PCR batch to monitor contamination. The amplified products were verified for size and specificity via 1.8% agarose gel electrophoresis and purified using the AMPure XP Beads Kit (Beckman Coulter, USA). The purified amplicons were then quantified using the Qubit 4.0 Fluorometer (Thermo Fisher Scientific, USA) and pooled equimolarly based on their concentrations to construct the sequencing library. Following standard protocols, the pooled library was subjected to paired-end sequencing (2 × 250 bp) on the Illumina MiSeq platform (Shanghai Biozeron Co., Ltd., China).

#### Data analysis

Raw sequencing data were preprocessed using a series of quality control steps to ensure the accuracy of downstream analyses. Initially, low-quality reads and adapter sequences were removed using Trimmomatic v0.33 with default parameters. Primer sequences were identified and trimmed using cutadapt v1.9.1 and custom Perl scripts, generating high-quality reads free from contamination. Assembled clean reads were obtained by merging overlapping paired-end reads with FLASH v1.2.7 under stringent conditions. Potential chimeric sequences were identified and removed using UCHIME v4.2, resulting in a final dataset of validated reads.^45^ Operational taxonomic units (OTUs) were clustered with a 97% similarity threshold using USEARCH v10 (http://drive5.com/uparse/).^46^ Representative sequences of each OTU were taxonomically classified against the SILVA 16S rRNA database using UCLUST v1.2.22q with a minimum confidence threshold of 0.8.^47^^,^^48^

Metrics such as Chao1, Abundance-based Coverage Estimator (Ace), and observed features were computed using Mothur (v1.30.1).^49^ Group comparisons for alpha diversity indices were performed using Tukey’s post-hoc test, with statistical significance set at *p* < 0.05. Weighted and unweighted UniFrac distances were calculated using QIIME (v1.7.0).^50^ Principal Coordinate Analysis (PCoA) and Non-metric Multidimensional Scaling (NMDS) were employed to visualize variations in community structure among groups. Statistical evaluation of clustering significance was performed using Adonis tests within the vegan package (v2.5-7) in R (v4.1.0). Venn diagrams depicting shared and unique OTUs among groups were generated using the web-based tool “Draw Venn Diagrams” (http://bioinformatics.psb.ugent.be/webtools/Venn). Relative abundances of bacterial taxa at the phylum, family, and genus levels were computed using the phyloseq package (v1.36.0) in R. Taxonomic composition was visualized as stacked bar plots generated with ggplot2 (v3.3.5). Differentially abundant features between groups were identified using Linear Discriminant Analysis Effect Size (LEfSe). Features with an LDA score ≥3.0 and *p* ≤ 0.05 were considered significant.^51^ Functional metagenomic profiling was performed using the Tax4Fun software package. This tool maps OTUs to KEGG and COG terms to infer the gut microbiome’s functional pathways and metabolic capacities. Functional differences among groups were visualized and analyzed at the COG (Cluster of Orthologous Groups) and KEGG pathway levels. Boxplots showing gut microbial composition and function inter-group distances were analyzed using Student’s t-test. SourceTracker was used to assess the contribution (microbiome transmission) of the plant and soil microbiome to forest musk deer. The gut microbiome of semi-release and release forest musk deer was examined as sink, while those of the microbiome from captive forest musk deer, plants, and soil were assessed as sources.

### Quantification and statistical analysis

Principal Coordinate Analysis (PCoA) was conducted with 999 permutations using the function Adonis within the vegan package (Figures 1F, 1G, 2A, 2C, and S1D, S1E, S2D, S2E, S3D, S3E, and S3F). Comparative analysis of Pseudomonas proportions among groups was performed using the Wilcoxon rank-sum test (Figure 1E). Boxplots showing gut microbial composition and function inter-group distances were analyzed using Student’s t-test (Figures 2B, 2D, S3A, S3B, and S3C). Metrics such as Chao1, ACE, and observed features were computed using Mothur. Significance between groups was assessed using the Wilcoxon rank-sum test (Figures S1A, S1B, S1C, S2A, S2B, and S2C). Differentially abundant features between groups were identified using Linear Discriminant Analysis Effect Size (LEfSe), based on analysis of variance (ANOVA) and Wilcoxon rank-sum tests. Features with an LDA score ≥3.0 and *p* ≤ 0.05 were considered statistically significant (Figures S4A, S4B, S4C, S4D, S5A, and S5E). Functional differences between groups were visualized at both the COG and KEGG pathway levels using the Tax4Fun software package, with significance assessed by the Kruskal-Wallis test (Figures S5B, S5C, S5D, S5F, S5G, and S5H).

## Supplemental information

Document S1. Figures S1–S5 and Table S2

Table S1. The sample information in this study The Excel file containing additional data related to sample information is too large to fit in a PDF.
