# Supplementary material for: Diet and environment drive the convergence of gut microbiome in wild-released giant pandas and forest musk deer
Source: iScience. 2025 Jun 7;28(7):112837. doi: 10.1016/j.isci.2025.112837 (PMC12226362; doi:10.1016/j.isci.2025.112837)
Supplement: Document S1. Figures S1–S5 and Table S2 [file mmc1.pdf]

iScience, Volume *28*

Supplemental information

Diet and environment drive the convergence of gut microbiome in wild-released giant pandas and forest musk deer

Chenyi Gao, Qiuyu Huang, Xinru Yang, Xinyuan Cui, Kaizhi Wen, Yue Liu, Chenyan Wang, Qinlong Dai, Jiadong Xie, and Lifeng Zhu

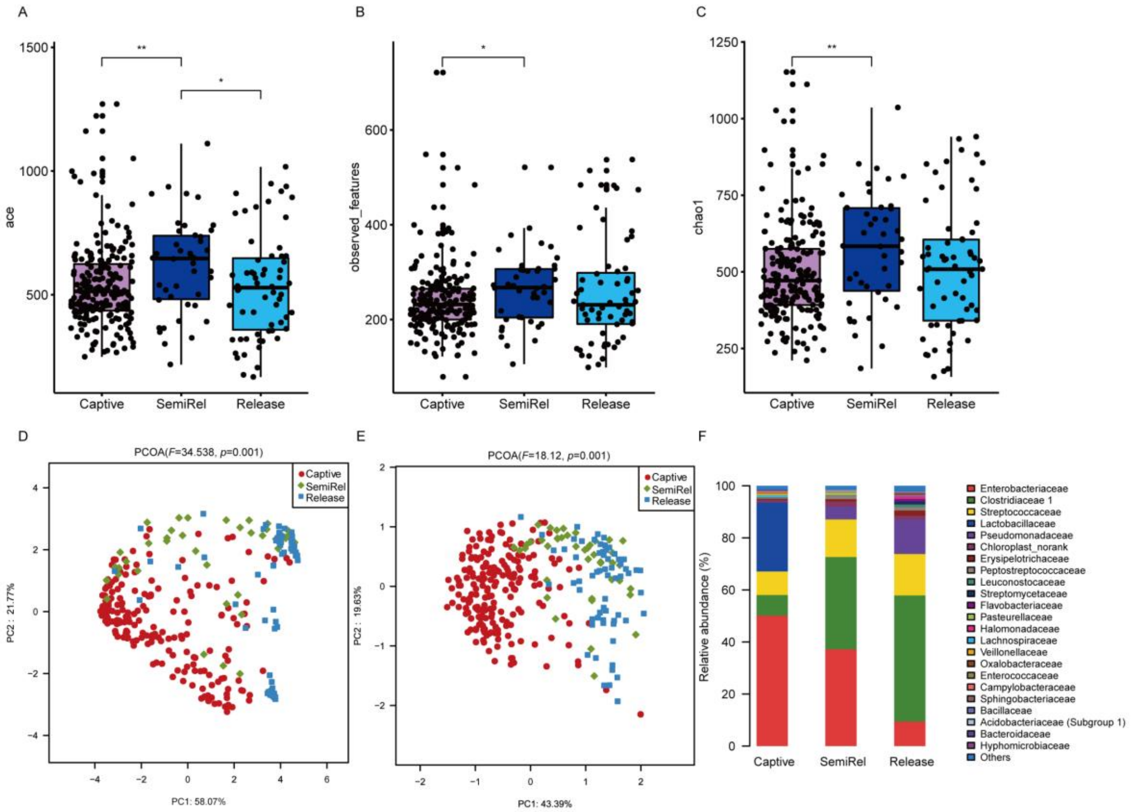

Figure S1. The Changes in the gut microbiome of giant pandas, related to Figure 1.

(A-C) Ace (Abundance-based Coverage Estimator), observed features, and chao1 richness to assess alpha diversity. Data are presented as the median value, lower quartile, and upper quartile, dots indicate discrete points. Wilcoxon rank-sum test, **p* < 0.05, ***p* < 0 .01.

(D and E) PCoA (Principal coordinates analysis) results based on Bray-Curtis and unweighted unifrac distances. PERMANOVA statistical analyses were conducted with 999 permutations using function Adonis.

(F) The dominant bacterial families in the gut microbiome of the three groups of giant pandas.

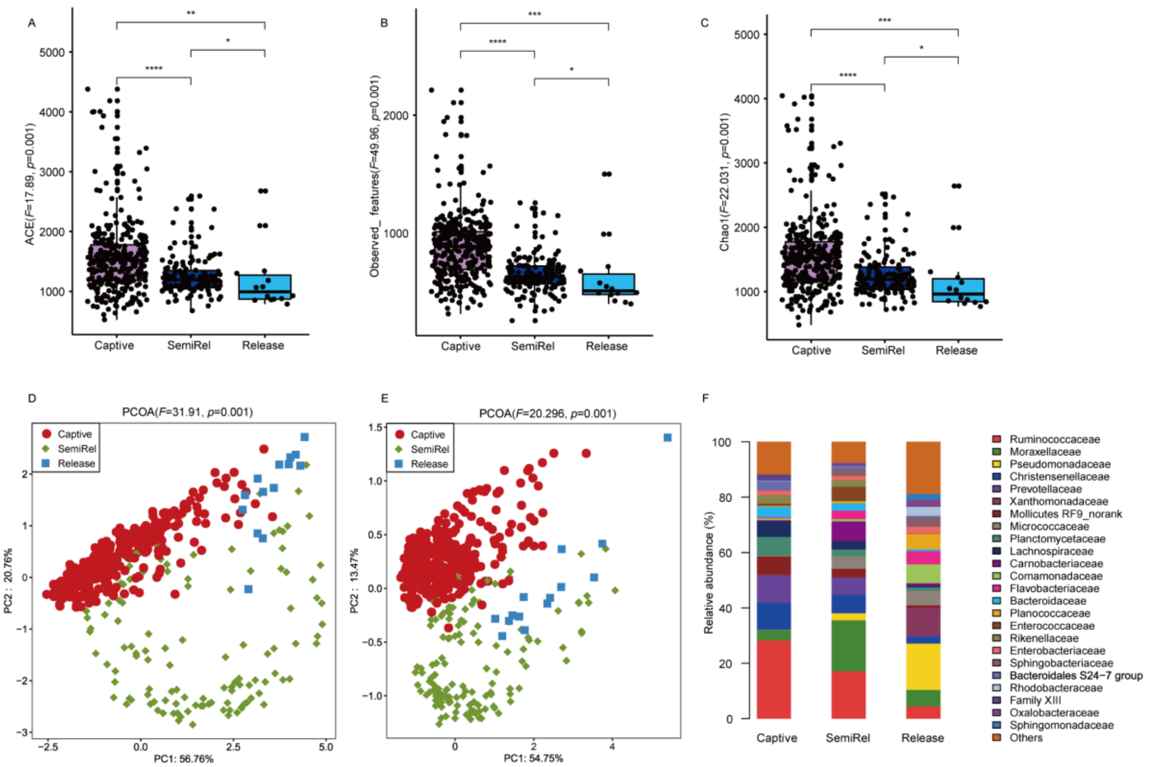

Figure S2. The Changes in the gut microbiome of forest musk deer, related to Figure 1.

(A-C) Ace, observed features, and chao1 richness to assess alpha diversity. Data are presented as the median value, lower quartile, and upper quartile, dots indicate discrete points. Wilcoxon rank-sum test, *p < 0.05, **p < 0 .01, *** p < 0 .001, ****p < 0 .0001.

(D and E) PCoA results based on Bray-Curtis and unweighted unifrac distances. PERMANOVA statistical analyses were conducted with 999 permutations using function Adonis.

(F)The dominant bacterial families in the gut microbiome of the three groups of forest musk deer.

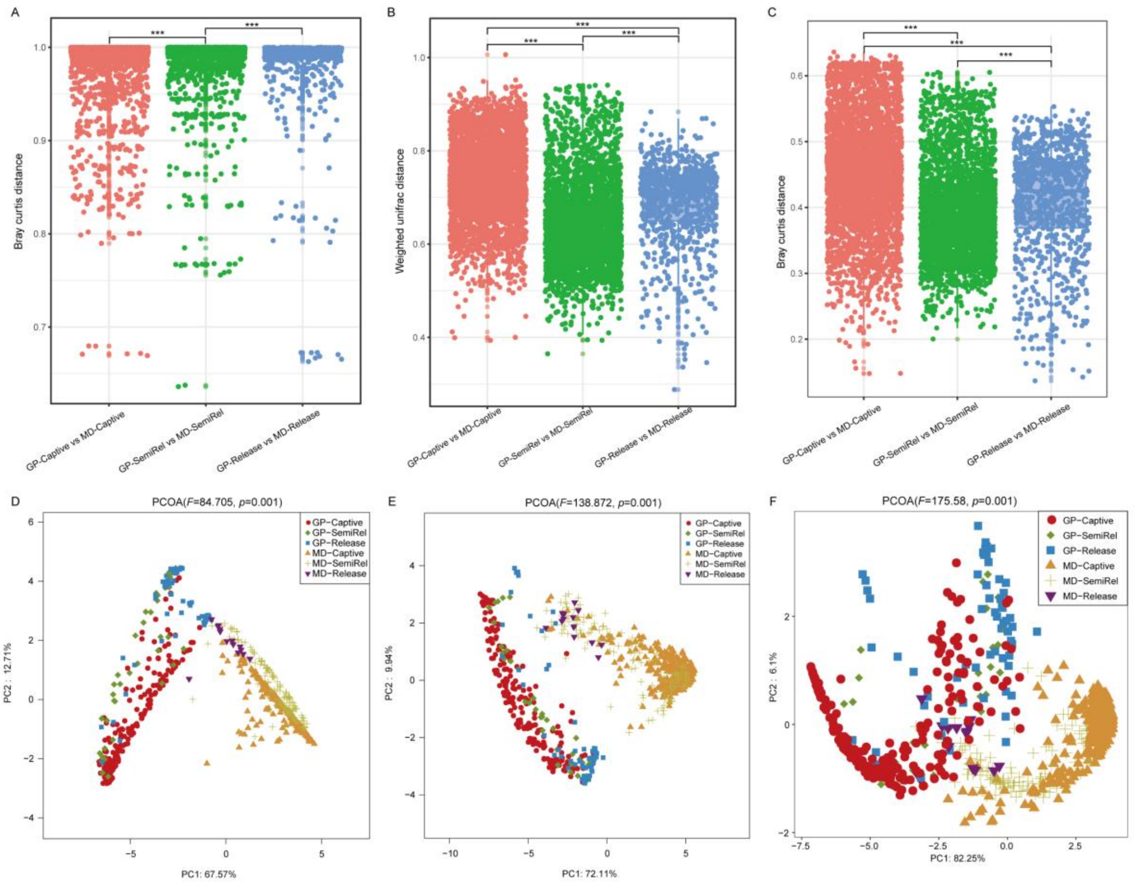

Figure S3. Convergent evolution of gut microbiome in giant pandas and forest musk deer, related to Figure 2.

(A and B) Comparative analysis of diversity based on Bray-Curtis and weighted unifrac distances at the composition level. Student's t-test, ****p* < 0.001.

(C) Comparative analysis based on Bray-Curtis distances at the KEGG function level. Student's t-test, ****p* < 0.001.

(D and E) PCoA results based on Bray-Curtis and weighted unifrac distances at the composition level. PERMANOVA statistical analyses were conducted with 999 permutations using function Adonis.

(F) PCoA results based on Bray-Curtis at the KEGG function level. PERMANOVA statistical analyses were conducted with 999 permutations using function Adonis.

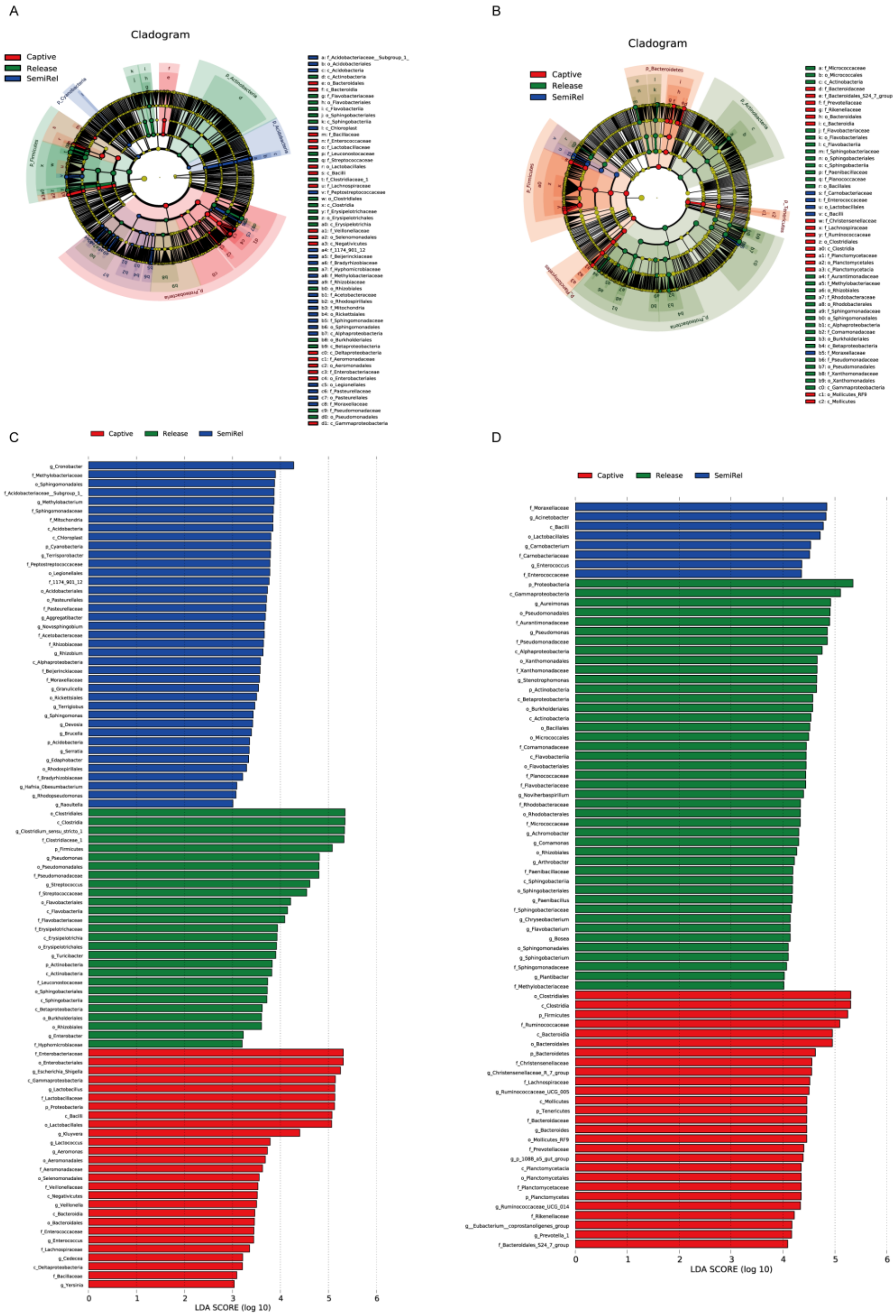

Figure S4. Differential analysis of gut microbiome between giant pandas and forest musk deer, related to Figure 1. Analysis of variance and Wilcoxon rank-sum test.

(A and B) LEfSe (Linear discriminant analysis Effect Size) was used to determine the significant difference in the abundance of gut microbiome of giant pandas and forest musk deer.

(C and D) Histogram of LDA values of gut differential species of captive, semi-release, and release of giant pandas and forest musk deer. (LDA value ≥ 3.0, $p \leqslant 0.05$)

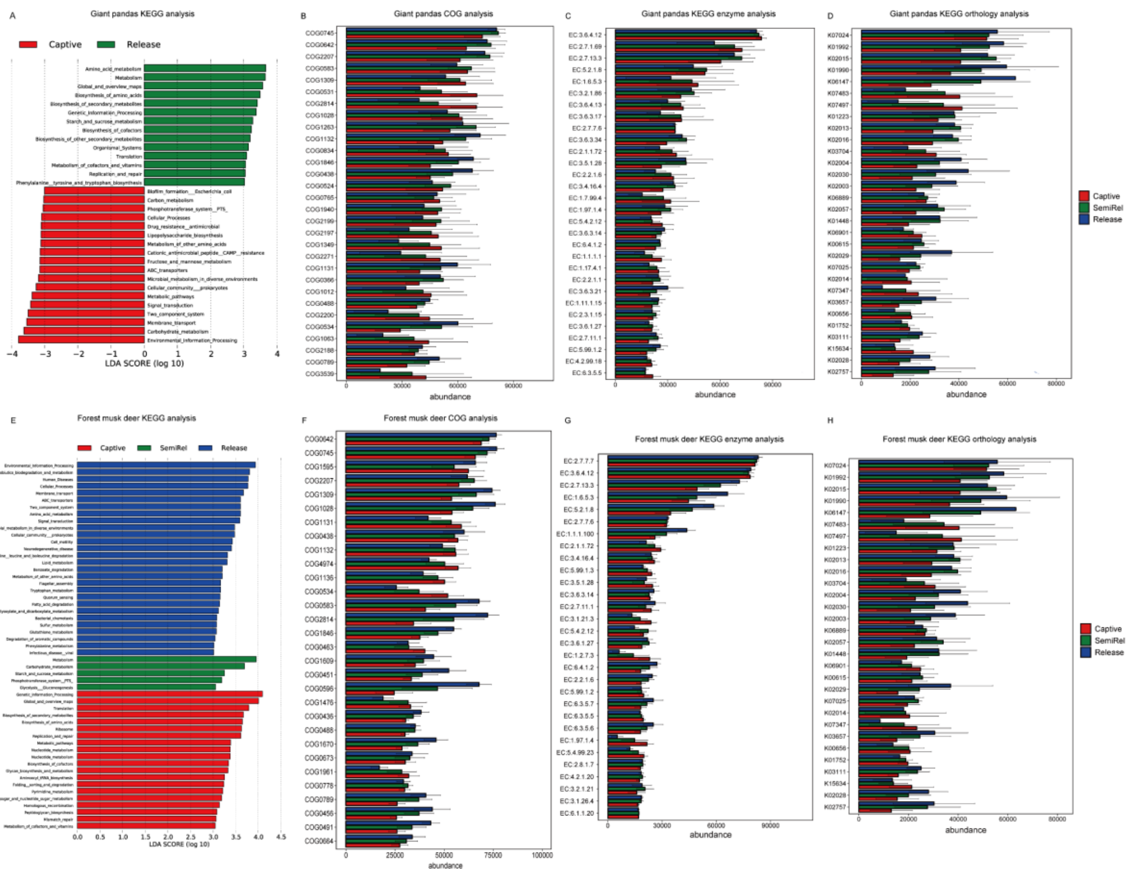

Figure S5. KEGG and COG enrichment analysis of giant pandas and forest musk deer, related to Figure 4.

(A and E) Giant pandas and forest musk deer analysis at the KEGG function level. Analysis of variance and Wilcoxon rank-sum test.

(B and F) Giant pandas and forest musk deer top 30 different functions at the COG function level. Kruskal-Wallis test.

(C and G) Giant pandas and forest musk deer top 30 different functions at the KEGG enzyme function level. Kruskal-Wallis test.

(D and H) Giant pandas and forest musk deer top 30 different functions at the KEGG orthology function level. Kruskal-Wallis test.

Table S2 plant and soil information

Sample ID	Species	sample source
Plant1	plant	in this study
Plant2	plant	in this study
Plant1	plant	in this study
Plant2	plant	in this study
Plant3	plant	in this study
Plant4	plant	in this study
Plant5	plant	in this study
Plant6	plant	in this study
Plant7	plant	in this study
Plant8	plant	in this study
Plant9	plant	in this study
Plant10	plant	in this study
Plant11	plant	in this study
Plant12	plant	in this study
Plant13	plant	in this study
Plant14	plant	in this study
Plant15	plant	in this study
Plant16	plant	in this study
Plant17	plant	in this study
Plant18	plant	in this study
Plant19	plant	in this study
Plant20	plant	in this study
Plant21	plant	in this study
Soil1	soil	in this study
Soil2	soil	in this study
Soil3	soil	in this study
Soil4	soil	in this study
Soil5	soil	in this study
Soil6	soil	in this study
Soil7	soil	in this study
Soil8	soil	in this study
Soil9	soil	in this study
Soil10	soil	in this study
Soil11	soil	in this study
Soil12	soil	in this study
Soil13	soil	in this study
Soil14	soil	in this study
Soil15	soil	in this study
Soil16	soil	in this study

Soil17	soil	in this study
Soil18	soil	in this study
Soil19	soil	in this study
Soil20	soil	in this study
Soil21	soil	in this study
Soil22	soil	in this study
Soil23	soil	in this study
